# A Targeted Mindfulness Curriculum for Medical Students During Their Emergency Medicine Clerkship Experience

**DOI:** 10.5811/westjem.2018.4.37018

**Published:** 2018-05-15

**Authors:** Arlene S. Chung, Rachel Felber, Ethan Han, Tina Mathew, Katie Rebillot, Antonios Likourezos

**Affiliations:** * Icahn School of Medicine at Mount Sinai, Department of Emergency Medicine, New York, New York; † Eisenhower Medical Center, Department of Emergency Medicine, Rancho Mirage, California; ‡ Lawnwood Regional Medical Center, Department of Emergency Medicine, Fort Pierce, Florida; § Harbor-UCLA, Department of Emergency Medicine, Torrance, California; ¶ Maimonides Medical Center, Department of Emergency Medicine, Brooklyn, New York

## Abstract

**Introduction:**

Despite high rates of burnout in senior medical students, many schools provide the majority of their wellness training during the first and second preclinical years. Students planning a career in emergency medicine (EM) may be at particularly high risk of burnout, given that EM has one of the highest burnout rates of all the specialties in the United States We developed an innovative, mindfulness-based curriculum designed to be integrated into a standard EM clerkship for senior medical students to help students manage stress and reduce their risk of burnout.

**Methods:**

The curriculum included these components: (1) four, once-weekly, 60-minute classroom sessions; (2) prerequisite reading assignments; (3) individual daily meditation practice and journaling; and (4) the development of a personalized wellness plan with the help of a mentor. The design was based on self-directed learning theory and focused on building relatedness, competence, and autonomy to help cultivate mindfulness.

**Results:**

Thirty students participated in the curriculum; 20 were included in the final analysis. Each student completed surveys prior to, immediately after, and six months after participation in the curriculum. We found significant changes in the self-reported behaviors and attitudes of the students immediately following participation in the curriculum, which were sustained up to six months later.

**Conclusion:**

Although this was a pilot study, our pilot curriculum had a significantly sustained self-reported behavioral impact on our students. In the future, this intervention could easily be adapted for any four-week rotation during medical school to reduce burnout and increase physician wellness.

## INTRODUCTION

Recent studies have demonstrated the high prevalence of burnout in residents and medical students.^1^,^2^ These unfortunate numbers have prompted medical educators to develop and implement new strategies to promote wellbeing and reduce stress. Of the different approaches, the most promising interventions focus on a concept known as mindfulness training. Originally developed in 1979 by Jon Kabat-Zinn, mindfulness-based stress reduction techniques have since been widely used and modified. Mindfulness programs for medical students generally consist of semester-long, elective courses during the pre-clinical years.^3^ Topics include gaining self-awareness, managing stress, and handling difficult emotions. Mindfulness is thought to decrease emotional reactivity through the act of paying attention to one’s thoughts and feelings. Meditation acts as a focused method for practicing the different aspects of mindfulness.

Students planning a career in emergency medicine (EM) may benefit from mindfulness training, as EM has one of the highest rates of burnout.^4^,^5^ Medical students who learn these skills may be uniquely positioned to succeed in residency and beyond. To the best of our knowledge, this is the first mindfulness-based curriculum designed for medical students participating in an EM clerkship.

## METHODS

In order to integrate this curriculum into a four-week EM clerkship, we developed four, once-weekly, 60-minute classroom sessions supplemented by reading assignments, individual meditation practice and journaling, and a wellness plan with the help of a mentor (Table 1). The full curriculum is included in Appendix A.

Students had readings and videos to review prior to each classroom session, as well as a short, weekly written assignment. The classroom sessions included techniques to encourage participation and collaborative learning, including the following: 1) ice breakers to build community; 2) brainstorming about the students’ current stressors to create relevance; 3) brief didactics about wellness and mindfulness to convey knowledge; 4) role-playing to foster value; and 5) practice exercises in mindfulness and meditation to develop competence. To build the value and skills required for sustained behavioral change, the students also regularly practiced meditation and tracked their progress via a daily practice journal that was reviewed weekly by a faculty mentor. The final assignment consisted of developing an individualized wellness plan using mindfulness-based techniques that the student could use following the completion of the curriculum.

A single faculty member (A.S.C.) led all of the classroom sessions, checked the daily practice journal, and worked with each student to develop his or her own individualized wellness plan. The faculty leader trained EM residents at the same institution (R.F., E.H., K.R.) to help facilitate group discussions and in-session activities. The faculty leader was not involved in determining the final grades for the students’ clerkships and did not participate in writing any standardized letters of evaluation for the students’ applications for residency programs.

Measured outcomes included surveys completed by the participants at baseline prior to starting the curriculum, immediately following the end of the four weeks, and again at six months. Each survey assessed for self-reported behaviors and attitudes regarding meditation and mindfulness and overall reactions to participation in the curriculum. Paired sample t-test analysis comparing baseline results against the results at four weeks and at six months was performed using standard software (SPSS©) with two-tailed statistical significance predetermined at p<0.05.

This pilot study was approved by the Maimonides Medical Center Institutional Review Board.

## RESULTS

We enrolled 30 students during three consecutive EM clerkship rotations at a single, urban, academic institution. EM is not a required clerkship at our institution and we preferentially select students for our clerkship who are planning a career in EM during the summer months of June, July, and August. Each student completed surveys prior to, immediately following, and six months after completing the curriculum. We excluded 10 students from the final analysis for failure to complete all three surveys. Responses were defined on a Likert scale as the following: 1=not at all/never; 2=a little/occasionally; 3=a lot/once at week; 4=very much/every day.

We found significant changes in the self-reported behaviors and attitudes of the students immediately following participation in the curriculum compared to prior to the curriculum (Table 2). Students believed more strongly in the importance of wellness for students and residents (p=0.01). They felt more confident that they could explain to another person how to meditate (p=0.0001) and be mindful (p=0.0001); more confident in their own ability to meditate (p=0.0001) and be mindful (p=0.0001); reported meditating more often (p=0.0001) and practicing mindfulness more often (p=0.0001); and were more likely to recommend meditation (p=0.0001) and mindfulness (p=0.0001) to another person.

More importantly however, many of these changes remained sustained at six months later (Table 2). Six months following participation, the students still felt more confident that they could explain to another person how to meditate (p=0.0001) and be mindful (p=0.0001); more confident in their own ability to meditate (p=0.012) and be mindful (p=0.0001); reported meditating more often (p=0.005) and practicing mindfulness more often (p=0.007); and were more likely to recommend meditation (p=0.008) and mindfulness (p=0.042) to another person when compared to prior to their participation in the curriculum.

Interestingly, three-quarters of the students (15/20, 75%) reported using their individualized wellness plan at least occasionally even up to six months later. Most students reported talking about either meditation (17/20, 85%) or mindfulness (17/20, 85%) to at least one other person since participating in the curriculum.

Students overall had very positive reactions to their participation in the curriculum. The majority of students responded that the prerequisite assignments were interesting and informative (17/20, 85%), the content covered in the classroom sessions was useful (20/20, 100%), and that the format of the classroom sessions was effective (19/20, 95%). Interestingly, more than half responded that regularly practicing meditation was important to them (13/20, 65%) and that they planned on using their individual wellness plan in the future (12/20, 60%). Many students (17/20, 85%) responded that they would recommend this course to others.

## DISCUSSION

Although some medical schools have implemented mindfulness training into their formal curriculum, most of these interventions occur during the pre-clinical years.^3^ Studies have demonstrated that burnout and suicidality increase during the third and fourth years.^1^,^6^ A recent systematic review highlighted the effectiveness of mindfulness interventions for reducing medical student stress, depression, fatigue, and burnout.^7^ We felt that developing a mindfulness-based curriculum for students designed to be integrated into their clerkship rotations would be one means to address this need for wellness programs in the later years of medical school.

Many clerkships include weekly didactic sessions within which our curriculum can be easily integrated. The curriculum may be led by the clerkship director or another faculty member invested in student wellbeing. The required resources are minimal, and no special equipment is required. Our curriculum can be easily reproduced at different institutions and for different clerkships, not just EM.

We measured student reactions, attitudes, and self-reported learning and behaviors according to the first two levels of the Kirkpatrick Model, which describes the progressive effectiveness of educational interventions as reaction, learning, changes in behavior, and patient outcomes. Reactions to the curriculum were positive, with most students reporting that they would recommend this curriculum to others. Most importantly, however, the self-reported changes in attitudes, learning, and behavior remained sustained even up to six months later, which is perhaps the most remarkable and impactful significance of this intervention.

## LIMITATIONS

Clear limitations of our study include a small sample size and implementation only at a single institution. We also acknowledge possible participation bias. We chose three summer months when students interested in EM rotate on our EM clerkships. It is unclear if these students were more motivated to participate in the curriculum given the timing. We attempted to mitigate this effect by ensuring that the faculty leader (A.S.C.) was not involved in the students’ final grades, standardized letters of evaluation, or any other letters of recommendation. We also excluded 10 students from the final analysis, which may have represented a second source of participation bias. Unfortunately, we did not measure demographic factors or other variables that would have allowed us to determine if there was a systematic difference between the students who completed all three surveys and those who did not.

Another limitation was the lack of burnout assessment or other similar outcomes. After careful consideration, we decided that, given the pilot nature of our study, evaluating a change in burnout as a result of our curriculum would be outside of the scope of our investigation.

Finally, our main outcomes were measured using a non-validated survey instrument. However, the survey questions were reviewed internally by the study authors (A.S.C., R.F., E.H., K.R.), and we felt that the questions appropriately assessed self-reported behaviors and attitudes. The questions were also limited by their self-report nature; however, directly observing outcomes was outside the scope of this initial curriculum evaluation.

## CONCLUSION

Although this was a pilot study, our innovative mindfulness-based curriculum had a significantly sustained impact on the attitudes and self-reported behaviors our students. Our intervention could easily be adapted for any four-week rotation during medical school.

## Supplementary Information



## Figures and Tables

**Table 1:** Content outline of mindfulness curriculum with prerequisite assignments, session objectives, classroom methods, and timeline for the final project.

	Session one: the basics	Session two: practicing mindfulness	Session three: mindfulness in daily life	Session four: reflections on mindfulness
Prerequisite reading/videos	NY Times article Medscape Report Dan Harris video #1 60 Minutes video	Chapter 1: Why Zebras Don’t Get Ulcers Power of Concentration Dan Harris video #2	9 Mindfulness Rituals 13 Things Mindful People Do Every Day	TED Talk Fallacy of Chasing Work-Life Balance Mindful Training
Objectives	Define foundational concepts relevant to wellness and mindfulness Identify personal stressors or stressful situations Practice meditation using breath technique	Summarize evidence supporting benefits of mindfulness Persuade a (role-play) patient to try meditation as a stress-reduction technique Practice meditation using the body scan	Discuss different strategies to incorporate mindfulness into daily activities Identify barriers to mindfulness Practice mindful eating	Reflect on changes associated with regular meditation Illustrate how mindfulness can improve personal life and patient care Select a preferred method of mindful meditation
Classroom methods	Formal Presentation Foundational concepts, including wellness, stress, burnout, meditation, mindfulness, and the MBSR program Group Discussion Identify commonly occurring situations in their personal and professional life that trigger stressful thoughts or feelings In-Session Activity “Museum Tour” to explore the different interpretations of wellness, burnout, and mindfulness 2-Minute Meditation Breath technique	Formal Presentation Evidence supporting effectiveness of meditation and mindfulness, including physiologic as well as mental health changes Group Discussion Share experiences with meditation practice over the past week, both positive and negative In-Session Activity Role-play scenarios in which the student promotes mindfulness as a stress reduction technique to a patient 2-Minute Meditation Body scan technique	Formal Presentation Methods to incorporate mindfulness into daily activities Group Discussion Share mindful experiences from both the clinical setting and in personal life In-Session Activity Think-pair-share to brainstorm barriers to practicing mindfulness in both clinical practice and in daily activities 2-Minute Meditation Mindful eating	Formal Presentation Relationship between reflection, mindfulness, and life-long learning Group Discussion Reflect on any changes that have occurred following a regular meditation practice In-Session Activity In teams, create a concept map to illustrate how mindfulness can enhance both personal wellness and patient care 2-Minute Meditation Meditation of choice
Weekly assignment (due on day of session)	Identify at least 3 specific stressors or stressful situations that the student has personally experienced.	Summarize the first week of meditation practice using either bullet points or prose.	Describe a case in which mindfulness was used during a clinical encounter, preferably during the previous week.	Short reflection any changes noted after implementing a regular schedule of meditation or on incorporating mindfulness in daily life
Individual wellness plan (approximate timeline)	—	First draft of plan	Refine draft using feedback from mentor	Complete and sign plan

**Table 2:** Survey responses at baseline (prior to the curriculum), four weeks (immediately following the curriculum), and six months after completing the curriculum. Statistical significance (p<0.05) comparing values at baseline vs. four weeks and again comparing baseline versus six months has been denoted with an (*).

Question	Baseline	4 wk	6 mo
I believe in the importance of wellness for medical students and residents.	3.35	3.65*	3.45
I feel confident that I can explain to another person how to meditate.	1.85	2.95*	2.90*
I feel confident in my own ability to meditate.	2.00	2.80*	2.65*
On average, I meditate _____.	1.45	2.80*	2.15*
How likely are you to recommend meditation to another person?	2.10	2.80*	2.65*
I feel confident that I can explain to another person how to be mindful.	2.00	3.20*	2.85*
I feel confident in my own ability to be mindful.	2.35	3.10*	2.95*
On average, I practice mindfulness _____.	1.95	3.15*	2.65*
How likely are you to recommend mindfulness to another person?	2.30	3.35*	2.70*
