# Supplementary material for: A Targeted Mindfulness Curriculum for Medical Students During Their Emergency Medicine Clerkship Experience
Source: West J Emerg Med. 2018 May 15;19(4):762–6. doi: 10.5811/westjem.2018.4.37018 (PMC6040904; doi:10.5811/westjem.2018.4.37018)
Supplement: Supplementary file 1 [file wjem-19-762-s001.pdf]

# A Targeted Mindfulness Curriculum for Medical Students During Their Emergency Medicine Clerkship Experience

Arlene S. Chung, MD, MACM
Rachel Felber, MD
Ethan Han, MD
Tina Mathew, MD
Katie Rebillot, DO
Antonios Likourezos, MPH, MA

# TABLE OF CONTENTS

Curriculum Overview .......... 3

Syllabus .......... 4

Session One: The Basics .......... 5

Session Two: Practicing Mindfulness .......... 6

Session Three: Mindfulness in Daily Life .......... 7

Session Four: Reflections on Mindfulness .......... 8

Daily Practice Journal .......... 9

Individualized Wellness Plan .......... 10

Learner Evaluation .......... 11

Program Evaluation .......... 13

Faculty Development .......... 14

Mindfulness Resources .......... 15

# CURRICULUM OVERVIEW

**DESCRIPTION OF LEARNERS:** MS3 and MS4 medical students planning a career in Emergency Medicine, currently participating in a four-week Emergency Medicine rotation

**CONTENT OUTLINE WITH INSTRUCTIONAL OBJECTIVES:**

| | **Session One:**<br>*The Basics* | **Session Two:**<br>*Practicing Mindfulness* | **Session Three:**<br>*Mindfulness in Daily Life* | **Session Four:**<br>*Reflections on Mindfulness* |
|---|---|---|---|---|
| *Prerequisite Reading/Videos* | NY Times article<br>Medscape Report<br>Dan Harris video #1<br>60 Minutes video | Chapter 1: Why Zebras Don't Get Ulcers<br>Power of Concentration<br>Dan Harris video #2 | 9 Mindfulness Rituals<br>13 Things Mindful People Do Every Day | TED Talk<br>Fallacy of Chasing Work-Life Balance<br>Mindful Training |
| *Objectives* | • Define foundational concepts relevant to wellness and mindfulness<br>• Identify personal stressors or stressful situations<br>• Practice meditation using breath technique | • Summarize evidence supporting benefits of mindfulness<br>• Persuade a (role-play) patient to try meditation as a stress-reduction technique<br>• Practice meditation using the body scan | • Discuss different strategies to incorporate mindfulness into daily activities<br>• Identify barriers to mindfulness<br>• Practice mindful eating | • Reflect on changes associated with regular meditation<br>• Illustrate how mindfulness can improve personal life and patient care<br>• Select a preferred method of mindful meditation |
| *Classroom Methods* | **Formal Presentation**<br>Foundational concepts, including wellness, stress, burnout, meditation, mindfulness, and the MBSR program<br><br>**Group Discussion**<br>Identify commonly occurring situations in their personal and professional life that trigger stressful thoughts or feelings<br><br>**In-Session Activity**<br>"Museum Tour" to explore the different interpretations of wellness, burnout, and mindfulness<br><br>**2-Minute Meditation**<br>Breath technique | **Formal Presentation**<br>Evidence supporting effectiveness of meditation and mindfulness, including physiologic as well as mental health changes<br><br>**Group Discussion**<br>Share experiences with meditation practice over the past week, both positive and negative<br><br>**In-Session Activity**<br>Role-play scenarios in which the student promotes mindfulness as a stress reduction technique to a patient<br><br>**2-Minute Meditation**<br>Body scan technique | **Formal Presentation**<br>Methods to incorporate mindfulness into daily activities<br><br>**Group Discussion**<br>Share mindful experiences from both the clinical setting and in personal life<br><br>**In-Session Activity**<br>Think-pair-share to brainstorm barriers to practicing mindfulness in both clinical practice and in daily activities<br><br>**2-Minute Meditation**<br>Mindful eating | **Formal Presentation**<br>Relationship between reflection, mindfulness, and life-long learning<br><br>**Group Discussion**<br>Reflect on any changes that have occurred following a regular meditation practice<br><br>**In-Session Activity**<br>In teams, create a concept map to illustrate how mindfulness can enhance both personal wellness and patient care<br><br>**2-Minute Meditation**<br>Meditation of choice |
| *Weekly Assignment (Due on day of session)* | Identify at least 3 specific stressors or stressful situations that the student has personally experienced. | Summarize the first week of meditation practice using either bullet points or prose. | Describe a case in which mindfulness was used during a clinical encounter, preferably during the previous week. | Short reflection any changes noted after implementing a regular schedule of meditation or on incorporating mindfulness in daily life |
| *Individual Wellness Plan (Approx. timeline)* | — | First draft of plan | Refine draft using feedback from mentor | Complete and sign plan |

**EVALUATION PLAN:**

- Learner Evaluation (see page 11 for more details)
- Program Evaluation (see page 13 for more details)

# SYLLABUS

Welcome to [YOUR INSTITUTION HERE]!

During the next four weeks, we will introduce you to basic wellness concepts and provide you with concrete skills to help you to address any stressors that you may encounter during the remaining time in medical school, transition to residency, and beyond. This course will consist of four one-hour classroom sessions during your didactic day. You will have prerequisite readings and videos to review prior to each class, as well as a short written assignment. We think that you find these both interesting and enlightening. Finally, at the end of the course, you will leave here with an individualized wellness plan that will hopefully serve you well in the months and years to come. Looking forward to working with you!

Sincerely,
[CURRICULUM DIRECTOR][CONTACT INFORMATION]

**COURSE OBJECTIVES**

- Be able to explain foundational wellness concepts, such as stress, burnout, continuous partial attention, meditation, and mindfulness
- Practice a regular schedule of meditation
- Create a wellness plan using mindfulness-based strategies to address individual risk factors for developing burnout

**STUDENT EVALUATION**

This course is pass/fail only. Your grade will be taken into consideration when determining your overall performance during the clerkship, but will not be officially listed on your transcript as separate grade. You will be evaluated using the following measures listed below and must receive a "pass" on your performance for each. Grading rubrics are provided on the next page:

- Group participation
- Daily practice journal
- One-page reflection
- Individualized wellness plan

**PROGRAM EVALUATION**

In order to create the most effective program for you, we welcome your feedback! You will be asked to complete a short series of questions, both before and after the course, in order to determine if we are doing an effective job of giving you the skills to combat burnout. The scores for these evaluation forms will <u>not</u> be factored into your evaluation for the course.

---

**PREREQUISITE READINGS/VIDEOS FOR SESSION #1**

*(to be completed prior to [DATE HERE])*

- Read New York Times article, "Why Do Doctors Commit Suicide?"
- Review Medscape's Physician Lifestyle Report 2015
- Watch, "Dan Harris' Panic Attack (and Discovery of Meditation)"
- Watch, "60 Minutes: Anderson Cooper on Mindfulness"

**ASSIGNMENT #1**

*(to be completed prior to [DATE HERE] and submitted via email to: [CURRICULUM DIRECTOR])*

- Identify at least 3 specific stressors or stressful situations that you have personally experienced. May be written in either paragraph form (less than 150 words) or bullet points

## SESSION ONE: THE BASICS

In this introductory session, students will learn the foundational concepts of physician wellness and mindfulness. One of the most important goals of this session is to bring the students' affective level regarding wellness to at least the level of receiving, from what may potentially be an affective level of zero (see page 14 for a more detailed description of the affective domain). In terms of motivational theory, this session promotes value in three ways: (1) group discussion to generate a list of common stressors in the students' academic and personal lives and (2) an evidence-based lecture on the importance of physician wellness and the positive impact of mindfulness strategies. In order to promote efficacy, students will participate as part of a group in a two-minute guided meditation followed by a debriefing on the experience. By the end of this session, students will be able to list the three components of burnout as defined by Maslach, explain the concept of mindfulness, and identify factors in their own lives that trigger stressful thoughts or feelings.

**SESSION #1 OBJECTIVES:** After participation in the session learners should be better able to:
- Define foundational concepts relevant to wellness and mindfulness
- Identify personal stressors or stressful situations
- Practice meditation using breath technique

**INSTRUCTIONAL MATERIALS**
- Short Power Point presentation (if needed)
- Whiteboard, chalkboard, or other method of writing down ideas
- Learner reaction session evaluation forms

**PREREQUISITE ASSIGNMENTS**
*(to be completed prior to the start of the session [DATE HERE])*
- Read New York Times article, "Why Do Doctors Commit Suicide?"
- Review Medscape's Physician Lifestyle Report 2015
- Watch, "Dan Harris' Panic Attack (and Discovery of Meditation)"
- Watch, "60 Minutes: Anderson Cooper on Mindfulness"

**ASSIGNMENT #1**
*(to be completed prior to [DATE HERE] and submitted via email to: [CURRICULUM DIRECTOR])*
- Identify at least 3 specific stressors or stressful situations that you have personally experienced. May be written in either paragraph form (less than 150 words) or bullet points.

| Time | Segment | Description |
|---|---|---|
| 0-10 | Session Introduction | Introduce the session instructor and the participants; present the curriculum and session objectives, expectations, and evaluation methods |
| 10-20 | Group Discussion | Identify commonly occurring situations from the students' personal and professional lives that trigger stressful thoughts or feelings |
| 20-30 | Short Lecture | Present foundational concepts, including wellness, stress, burnout, meditation, mindfulness, and the MBSR program |
| 30-45 | "Museum Tour" | In three rotating groups, explore the different interpretations of wellness, burnout, and mindfulness |
| 45-55 | 2-Minute Meditation | Two minutes of guided meditation focused on breath, followed by group discussion of the experience |
| 55-60 | Session Closer | Review session objectives; discuss prerequisite assignments for next session; answer questions regarding the daily practice journal and creation of the individualized wellness plan; distribute evaluation forms to assess learners' reactions |

## SESSION TWO: PRACTICING MINDFULNESS

In this second session, students will learn the evidence supporting the effectiveness of mindfulness and meditation practice. The students will have engaged in one week of regular meditation practice by this time and may have experienced some of the positive effects on a personal level. They will also be given the opportunity to share any negative experiences as well. In terms of increasing the students' affective level, this session builds on the first session by having them role-play a scenario in which they promote mindfulness and meditation (see page 14 for a more detailed description of the affective domain). They will also be introduced to a second method of mindful meditation—the body scan technique. By the end of this session, students will be able to summarize the hard evidence supporting the benefits of mindfulness and feel more comfortable promoting mindfulness and meditation to patients.

**SESSION #2 OBJECTIVES**

- Summarize evidence supporting benefits of mindfulness
- Persuade a (role-play) patient to try meditation as a stress-reduction technique
- Practice meditation using the body scan

**INSTRUCTIONAL MATERIALS**

- Short Power Point presentation (if needed)
- Printed role play scenarios
- Learner reaction session evaluation forms

**PREREQUISITE ASSIGNMENTS**

*(to be completed prior to the start of the session [DATE HERE])*

- Read the New York Times article, "The Power of Concentration."
- Read an excerpt from *Why Zebras Don't Get Ulcers*, "Chapter 1: Why Don't Zebras Get Ulcers?"
- Watch "Dan Harris: Hack Your Brain's Default Mode with Meditation."

**ASSIGNMENT #2**

*(to be completed prior to [DATE HERE] and submitted via email to: [CURRICULUM DIRECTOR])*

- Summarize the first week of meditation practice using either bullet points or prose. Can include reflection, graphs or tables, and/or a discussion of recurring themes. Limit to 150 words or ½ page.

| Time | Segment | Description |
|---|---|---|
| 0 to 5 | Session Introduction | Present the session objectives; explain how this session fits into the larger curriculum |
| 5-15 | Group Discussion | Students share experiences with their individual meditation practice over the past week, both positive and negative |
| 15-25 | Short Lecture | Present evidence supporting the effectiveness of meditation and mindfulness, including physiologic and mental health changes |
| 25-40 | Role-play scenarios | Role-play scenarios in which the student promotes mindfulness as a stress reduction technique to a patient |
| 40-50 | 2-Minute Meditation | Two minutes of guided meditation using the body scan technique, followed by group discussion of the experience |
| 50-60 | Session Closer | Review session objectives; discuss prerequisite assignments for next session; answer questions regarding the daily practice journal and creation of the individualized wellness plan; distribute evaluation forms to assess learners' reactions |

## SESSION THREE: MINDFULNESS IN DAILY LIFE

The purpose of this third session is to help the students to incorporate mindfulness into everyday activities. Up until this point, the emphasis has been on practicing meditation and mindfulness in a quiet environment supportive to this type of mental activity. The challenge this week is to try to practice mindfulness "on-the-fly" so to speak. The formal presentation will provide them with different methods and examples of integrating mindfulness into different activities, both in the clinical arena and outside of it. Students will also share their own experiences during the previous week. The think-pair-share activity gives the students an opportunity to discuss their struggles more privately in pairs before engaging in the group discussion. The two-minute meditation this week, mindful eating, provides one concrete method of incorporating mindfulness into a common activity that recurs multiple times each day. By the end of this session, students will be able to identify their own barriers to practicing mindfulness and leave with a concrete list of ways to add more mindfulness in each day.

**SESSION #3 OBJECTIVES**

- Discuss different strategies to incorporate mindfulness into daily activities
- Identify barriers to mindfulness
- Practice mindful eating

**INSTRUCTIONAL MATERIALS**

- Short Power Point presentation (if needed)
- Index cards or sheets of paper and pens for the student to write
- A few boxes of raisins (or other food item)
- Learner reaction session evaluation forms

**PREREQUISITE ASSIGNMENTS**

*(to be completed prior to the start of the session [DATE HERE])*

- Read, "Nine Mindfulness Rituals to Make Your Day Better"
- Read, "Thirteen Things Mindful People Do Differently Every Day"

**ASSIGNMENT #3**

*(to be completed prior to [DATE HERE] and submitted via email to: [CURRICULUM DIRECTOR])*

- Describe a case (less than 150 words) in which you used mindfulness during a clinical encounter, preferably during the previous week. Can be your own use of mindfulness or promoting mindfulness to a patient or colleague.

| Time | Segment | Description |
|---|---|---|
| 0 to 5 | Session Introduction | Present the session objectives; explain how this session fits into the larger curriculum |
| 5-15 | Group Discussion | Students share mindful experiences from both the clinical setting and in personal life |
| 15-25 | Short Lecture | Present specific ways to incorporate mindfulness into daily activities |
| 25-40 | Think-Pair-Share | First in pairs, then as a group, students brainstorm barriers to practicing mindfulness in both clinical practice and in daily activities |
| 40-50 | 2-Minute Meditation | Two minutes of guided meditation using mindful eating, followed by group discussion of the experience |
| 50-60 | Session Closer | Review session objectives; discuss prerequisite assignments for next session; answer questions regarding the daily practice journal and creation of the individualized wellness plan; distribute evaluation forms to assess learners' reactions |

## SESSION FOUR: REFLECTIONS ON MINDFULNESS

*The unexamined life is not worth living.*
*–Socrates*

Reflection is a valuable method for self-directed learning and integrating new information with prior knowledge. This final session focuses on helping the students to reflect on the previous four weeks. They have the opportunity to reflect individually for their written assignment due for the session as well as to share their thoughts verbally with the group. Creation of a concept map requires not just knowledge of the foundational concepts, but also the ability to analyze and generate connections. Understanding more connections between wellness, mindfulness, personal happiness, and patient care will hopefully strengthen the knowledge, skills, and attitudes that the students have gained during their participation in the course. By the end of this session, students will have spent time reflecting on their own practice of meditation and everyday mindfulness with the goal of at least recognizing the value of these skills going forward.

**SESSION #4 OBJECTIVES**
- Reflect on changes associated with regular meditation
- Illustrate how mindfulness can improve personal life and patient care
- Select a preferred method of mindful meditation

**INSTRUCTIONAL MATERIALS**
- Short Power Point presentation (if needed)
- Whiteboard, chalkboard, or other method of writing down ideas
- Learner reaction session evaluation forms
- Post-curriculum survey assessing knowledge, skills, attitudes, as well as the two-item MBI for burnout

**PREREQUISITE ASSIGNMENTS**
*(to be completed prior to the start of the session [DATE HERE])*
- Watch the TED Talk, "Andy Puddicombe: All It Takes is 10 Mindful Minutes"
- Read, "The Fallacy of Chasing After Work-Life Balance"

**ASSIGNMENT #4**
*(to be completed prior to [DATE HERE] and submitted via email to: [CURRICULUM DIRECTOR])*
- Short (approx. 300 words) reflection on any changes noted after implementing a regular schedule of meditation or on incorporating mindfulness in daily life

| Time | Segment | Description |
|---|---|---|
| 0 to 5 | Session Introduction | Present the session objectives; explain how this session fits into the larger curriculum |
| 5-15 | Group Discussion | Students reflect on any changes that have occurred following a regular meditation practice |
| 15-25 | Short Lecture | Relationship between reflection, mindfulness, and life-long learning |
| 25-40 | Concept Map | In teams of three or four, students create a concept map to illustrate how mindfulness can enhance both personal wellness and patient care |
| 40-45 | 2-Minute Meditation | Two-minute meditation of choice |
| 45-60 | Session Closer | Review session and curriculum objectives; answer any final questions about the individualized wellness plan; discuss six-month follow-up; distribute short survey assessing knowledge, skills, attitudes, as well as the two-item MBI for burnout |

## DAILY PRACTICE JOURNAL

**DESCRIPTION:** The daily practice journal is a Google Document that is shared with the curriculum director that includes space for the date, time, duration of meditation, method used (e.g. breath, sound, body scan, mindful eating), location of practice, and comments (e.g. thoughts during and after, degree of difficulty maintaining concentration). There is also space for recording reasons for any missed sessions. The practice journal will appear as in the example below (the first four days of the rotation have been completed by the student in this example).

**RELEVANT COMPETENCY:** Professionalism

**RATIONALE:** Using an electronic format rather than traditional paper allows for real-time sharing of information between the student and curriculum director.

**EVALUATION:** See “Learner Evaluation” on page 12

---

## MINDFULNESS IN EMERGENCY MEDICINE

### Daily Practice Journal

**INSTRUCTIONS:** Fill out the table below for each day. Include the time of day, duration of the meditation session, where it took place, and the method used. Feel free to practice in any way that feels comfortable for you. You may practice more than once each day. Moments of mindfulness during the day count as meditation sessions as well. Record any notable thoughts, distractions, or themes in the notes column. Write with as much or as little detail as you like. If you miss a day, simply write “missed” in the notes column.

**REQUIREMENTS:** You are expected to practice at least 75% of the days of the rotation (21 out of 28 days). [CURRICULUM DIRECTOR] will regularly review your journal to track your progress.

If you have any questions, feel free to contact [CURRICULUM DIRECTOR][CONTACT INFORMATION].

Happy meditating!

| Date | Time | Duration | Location | Method | Notes |
|---|---|---|---|---|---|
| 7/1 | 9:30PM | 2 min | My room | Breathing | Was really hard to concentrate, kept thinking about how I was really late this morning, also was really tired |
| 7/2 | | | | | MISSED |
| 7/3 | | | | | MISSED |
| 7/4 | 8PM | 2 min | My room | Body scan | Easier to focus with body scan |
| 7/5 | | | | | |
| 7/6 | | | | | |
| 7/7 | | | | | |

## INDIVIDUALIZED WELLNESS PLAN

**DESCRIPTION:** A Word document completed by the student with assistance from the curriculum director. (The first line has been completed by the student in the example below.)

**RELEVANT COMPETENCY:** Professionalism, Practice-Based Learning Improvement

**RATIONALE:** Tasking the students to create his or her own individual wellness plan utilizes multiple learning principles. It promotes efficacy by giving the student concrete actions to combat stress; requires reflection and self-assessment to identify personal stressors and potential barriers; and fosters life-long learning principles by having the student, rather than the instructor, complete the plan.

**EVALUATION:** See "Learner Evaluation" on page 12

---

## MINDFULNESS IN EMERGENCY MEDICINE

### Individual Wellness Plan

**INSTRUCTIONS:** This is your personal wellness plan that has been designed to help you during times of stress in the coming months. You may fill out the table in any order that you wish, although we recommend that you complete the "stressor" and "reason" columns after the first classroom session (The Basics) and the "potential barriers," "action plan," and "countermeasures" columns after the third classroom session (Mindfulness in Daily Life).

**REQUIREMENTS:** A first draft of your plan should be submitted to [CURRICULUM DIRECTOR][CONTACT INFORMATION] by the end of the third week, in order to receive feedback. You are expected to complete the "final" version of your wellness plan by the end of your Emergency Medicine rotation (although we very much encourage you to continue to add to it as needed once you leave us). You will need both signatures below to complete the plan.

| STRESSOR (WHAT) | REASON (WHY) | ACTION PLAN (HOW) | POTENTIAL BARRIERS | COUNTER MEASURES |
|---|---|---|---|---|
| Step 2 CK | Didn't get a high enough score on Step I and worried that it might affect my interviews/match list | When feeling overwhelmed by how much I need to study, take one minute to focus on breathing | Forgetting my action plan | Leave a sticky note above my desk that says "Remember to breathe" |
| | | | | |
| | | | | |
| | | | | |

By typing or signing my name below, I hereby certify that I will follow the action plan and countermeasures outlined above to the best of my abilities <u>because I care about my own personal wellness</u>.

**STUDENT SIGNATURE** ____________________ **DATE**__________

**CURRICULUM DIRECTOR SIGNATURE** ____________________ **DATE**__________

# LEARNER EVALUATION

Evaluation of the learners occurs in two different ways. At its most basic, the learners will be assessed on essentially whether or not they completed the requirements of the course to a satisfactory degree. This is their "pass/fail grade" for the course. More importantly however, the learners will also be assessed on the first three levels of Kirkpatrick's Model of Educational Outcomes, which is critical for determining the impact and effectiveness of the curriculum.

## PASS/FAIL GRADE

This course is pass/fail only. The grade for this course will be taken into consideration when determining the student's overall performance during the clerkship but will not be officially listed on his or her transcript as separate grade. The clerkship director remains responsible for the final grade received by the student for the rotation that will appear on the transcript.

The student must receive a "pass" on his or her performance for all of the following:

- Group participation
- Daily practice journal
- Short reflection
- Individualized wellness plan

**GROUP PARTICIPATION:** *Formative Assessments*—If a student is noted to be either unprepared or disrespectful during a session, it is the responsibility of the curriculum director to bring the inappropriate behavior to the attention of the student in a timely manner. *Summative Assessment*—According to the rubric below:

| FAIL | PASS | |
|---|---|---|
| *Below expectations* | *Meets expectations* | *Above expectations* |
| Unprepared for discussions (unable to summarize the content of the required readings and videos). | Prepared for discussions (able to summarize the content of the required readings and videos). | Performs own literature search prior to discussions and brings in new readings and resources to discuss. |
| Openly disrespectful of others' opinions. Does not maintain others' privacy. | Respectful of others' opinions and maintains their privacy. | Sensitively explores others' viewpoints while maintaining respect for their privacy and personal feelings. |

**DAILY PRACTICE JOURNAL:** *Formative Assessments*—The curriculum director will review each student's practice journal at the end of each week. If he or she falls below the projected 75% of days expected for meditation or mindfulness practice, the student will be contacted via email. It is up to the discretion of the curriculum director whether or not to arrange a face-to-face meeting to address any barriers to practice. *Summative Assessment*—According to the rubric below:

| FAIL | PASS | |
|---|---|---|
| *Below expectations* | *Meets expectations* | *Above expectations* |
| Practices < 75% of days | Practices at least 75% of days | Practices daily |
| Does not record any thoughts or reflections following each session | Records basic thoughts that occurred during each session. Reflects on any external factors that may have contributed to the success or difficulty of the session (e.g. lack of sleep, time of day, location of practice) | Records detailed thoughts that occurred during each session, notes common themes and/or problems that have occurred across multiple sessions, discusses possible solutions to any problems |

## LEARNER EVALUATION (cont.)

**REFLECTION GRADING RUBRIC:** *Summative Assessment Only*—According to the rubric below. In addition, the curriculum director will provide written or verbal feedback to the student about their reflection.

| FAIL | PASS | |
|---|---|---|
| *Below expectations* | *Meets expectations* | *Above expectations* |
| Does not respond to the assignment. | Describes with some reflecting and superficial discussion of lessons learned | Integrates prior experience, provides justification of lessons learned, suggests further action |

**INDIVIDUALIZED WELLNESS PLAN:** *Formative Assessment*—Students should complete a first draft of their wellness plan by the end of the third week, following Session Three (Mindfulness in Daily Life). This will allow the curriculum director time to review the plan and offer suggestions. The students are also welcome to submit multiple drafts at any point for review. *Summative Assessment*—According to the rubric below:

| FAIL | PASS | |
|---|---|---|
| *Below expectations* | *Meets expectations* | *Above expectations* |
| Does not complete assignment | Completes all columns for at least 3 specific stressors | Completes all columns for more than 3 specific stressors |
| No reasoning and non-actionable plans and countermeasures | Basic reasoning and acceptable, but somewhat vague plans and countermeasures | Thoughtful reasoning and specific action plans and countermeasures |

### KIRKPATRICK'S MODEL OF EDUCATIONAL OUTCOMES

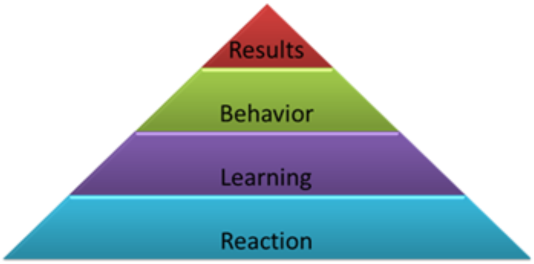


**LEVEL 1 (Reaction):** Following each classroom session, students will complete a short evaluation form to assess their reaction to the prerequisite assignments, classroom activities, facilitator skill, and written assignment. Their degree of group participation will also be assessed according to the rubric above.

**LEVEL 2 (Learning):** Students will complete a short multiple-choice knowledge exam covering the foundational concepts of wellness, burnout, mindfulness, and meditation before and after the curriculum to determine any increase in knowledge. At the end of this exam, they will also complete an abbreviated two-item MBI, rate their attitudes toward meditation and mindfulness, and their own perceived skill at meditation and mindfulness, both before and after the curriculum.

**LEVEL 3 (Behavior):** Six months following completion of the curriculum, the students will be contacted via email and/or phone interviews to assess the degree of adherence to their wellness plan and continuation of meditation or mindfulness practice.

## PROGRAM EVALUATION

The ultimate goal of this curriculum is to provide medical students planning on a career in Emergency Medicine with the knowledge, skills, attitudes, and behaviors to address their own personal stressors in order to reduce their risk for burnout in the future. Evaluation of this program therefore aims to determine to what degree this goal was accomplished.

**BURNOUT:** Each student will complete a two-item MBI form to determine their level of burnout before and after the curriculum. The two-item form has been adapted from the full 22-item original MBI and also found to be valid for determining burnout.

| | Strongly disagree | | | Neither agree nor disagree | | | Strongly agree |
|---|---|---|---|---|---|---|---|
| I feel burned out from my work | 1 | 2 | 3 | 4 | 5 | 6 | 7 |
| I have become more calloused toward people since I took this job | 1 | 2 | 3 | 4 | 5 | 6 | 7 |

West CP et al. "Single Item Measures of Emotional Exhaustion and Depersonalization Are Useful for Assessing Burnout in Medical Professionals." J Gen Intern Med. 2009 Dec; 24(12): 1318-1321.

**LEARNER REACTIONS:** As noted above, following each classroom session, students will complete a short evaluation form to assess their reaction to the prerequisite assignments, classroom activities, facilitator skill, and written assignment. This data will be used to improve any consistent program deficiencies as perceived by the learners.

**KNOWLEDGE:** As noted above, students will complete a short multiple-choice knowledge exam covering the foundational concepts of wellness, burnout, mindfulness, and meditation before and after the curriculum to determine any increase in knowledge. Any consistent gaps in knowledge will be specifically addressed in future revisions of the formal presentations for the relevant classroom session.

**SKILLS:** Students will practice a regular schedule meditation and mindfulness to increase their confidence and ability in these specific skills. As noted above, they will rate their own perceived skill at meditation and mindfulness, both before and after the curriculum. Each student's daily practice journal will also be reviewed as an additional source of data for the student's perceived ability level.

**ATTITUDES:** As noted above, students will rate their attitudes toward meditation and mindfulness both before and after the curriculum. Their attitudes will also be assessed during their participation in each of the classroom sessions. Session One aims to bring the students to at least an affective level of "receiving," if not "responding." In order to sustain behavioral change, learners must reach at least the level of "valuing," which Session Two attempts to accomplish via role-play scenarios. The last two classroom sessions hope to reinforce these attitudes.

**Taxonomy of the Affective Domain**

| *Category* | *Definition* |
|---|---|
| Receiving | Awareness, willingness to hear, selected attention |
| Responding | Active participant (amount, willingness, satisfaction) |
| Valuing | Willingness to be seen by others as holding the value |
| Organizing | Bringing new value into harmony with existing values |
| Characterizing | Internal regulation, consistent performance (does it without thinking about it) |

Krathwohl DR et al. Taxonomy of Educational Objectives: Handbook II: Affective Domain. New York: David McKay Co, 1964.

**BEHAVIORS:** As noted above, six months following completion of the curriculum, the students will be contacted via email and/or phone interviews to assess the degree of sustained adherence to their wellness plan and continuation of meditation or mindfulness practice.

# FACULTY DEVELOPMENT

Here is a more detailed description of some of the teaching techniques used throughout the mindfulness curriculum that may be unfamiliar to some educators

**FACILITATED DISCUSSION:** Facilitation is used to enhance the effectiveness of small and large group discussions, to assist learners to gain personal awareness in relation to any issue, and to assist learners in effective interactions with each other so that they learn from each other. In order to facilitate effectively, prepare yourself intellectually (read and reflect prior to the session) and emotionally (remind yourself to listen and encourage; not react). Facilitation works best in an open atmosphere, where learners feel comfortable sharing their ideas, which typically requires first establishing and reinforcing ground rules for discussion that focus on careful listening and respectful talking. Lastly, do not allow the discussion to degenerate into successive dialogues with the facilitator (or worse, a facilitator monologue).

**"MUSEUM TOUR":** A "museum tour" is done by having each group generate a list of answers in response to a prompt, for example: "Wellness—definitions," "Burnout—definitions," and "Mindfulness—definitions." The groups then rotate by one, so that the group that initially generated the list for wellness now adds to the list for burnout, etc. The groups then switch two more times, such that that end up in front of their initial list but are now able to view the comments added by the other two groups.

**THINK-PAIR-SHARE:** This is a rapid method to engage a medium sized or large audience in thinking about a topic of interest, particularly sensitive arenas. Learners are given a stimulus (e.g. a question) and asked to think for a minute or two, write down their thoughts either as bullet points or as complete sentences. This is typically followed by the opportunity to share with a partner (full participation) and then the pair can volunteer to share with the entire group (partial participation).

**CONCEPT MAPPING:** A concept map is essentially a visual representation of the multiple connections between different concepts or ideas. Not surprisingly, it is most useful for exploring complex topics. There is no "right way" to diagram a concept map and each person may interpret the connections between similar topics in different ways.

**BREATH MEDITATION:** Sit comfortably and close your eyes. Now bring the attention to the breath. Simply notice the breath as it moves in and out as the body inhales and exhales. Notice how the breath moves in and out automatically, effortlessly. Don't try to manipulate it in any way. Notice all the details of the experience of breathing—the feeling of the air moving in and out of the nose and the way the body moves as it breathes.

**BODY SCAN MEDITATION:** This involves systematically sweeping through the body with the mind, bringing an affectionate, open-hearted, interested attention to its various regions, customarily starting from the toes of the left foot to the top of the head.

**MINDFUL EATING:** This example uses a raisin, although any food can be substituted. First hold the raisin and take time to notice all the visual details of the different folds and hollows. Explore the texture of the raisin with your fingertips. Smell the raisin. Place the raisin in your mouth and hold it there without chewing, noting the sensation as you explore it with your tongue. When you are ready, take one or two bites and notice the waves of taste that emanate from it and how these change over time. Swallow, feeling the sensation. Finally sit and feel, noting how your body feels one raisin heavier.

# MINDFULNESS RESOURCES

**SESSION ONE: The Basics**

- Sinha P. Why Do Doctors Commit Suicide? New York Times. Sep 4, 2014. Available at: http://www.nytimes.com/2014/09/05/opinion/why-do-doctors-commit-suicide.html?_r=0
- Peckham C. Physician Lifestyle Report 2015. Medscape. Available at: http://www.medscape.com/features/slideshow/lifestyle/2015/public/overview#6
- Dan Harris' Panic Attack (and Discovery of Meditation). Available at: http://bigthink.com/think-tank/meditation-2
- Anderson Cooper on Mindfulness. 60 Minutes. Available at: http://www.cbsnews.com/news/mindfulness-anderson-cooper-60-minutes/

**SESSION TWO: Practicing Mindfulness**

- Konnikova M. The Power of Concentration. New York Times. Dec 15, 2012. Available at: http://www.nytimes.com/2012/12/16/opinion/sunday/the-power-of-concentration.html?pagewanted=all&_r=0
- Sapolsky RM. Chapter 1: Why Don't Zebras Get Ulcers? pp. 1-8. In: *Why Zebras Don't Get Ulcers*.
- Dan Harris: Hack Your Brain's Default Mode with Meditation. Available on YouTube at: https://www.youtube.com/watch?v=FAcTIrA2Qhk

**SESSION THREE: Mindfulness in Daily Life**

- Babauta L. Nine Mindfulness Rituals to Make Your Day Better. Available at: http://zenhabits.net/ritual/
- Gregoire C. Thirteen Things Mindful People Do Differently Every day. Available at: http://www.huffingtonpost.com/2014/04/30/habits-mindful-people_n_5186510.html

**SESSION FOUR: Reflections on Mindfulness**

- Andy Puddicombe: All It Takes is 10 Mindful Minutes. Available on TED Talks at: https://www.ted.com/talks/andy_puddicombe_all_it_takes_is_10_mindful_minutes
- Schwingshackl A. The Fallacy of Chasing After Work-Life Balance. Frontiers in Pediatrics. Mar 31, 2014. Available at: http://journal.frontiersin.org/article/10.3389/fped.2014.00026/full

---

## WEBSITES

- Palouse Mindfulness: Stress Reduction and Wellness. Free online Mindfulness Based Stress Reduction Course. Available at: http://palousemindfulness.com/selfguidedMBSR.html
- American College of Emergency Physicians Wellness Section and Resources. Available at: http://www.acep.org/content.aspx?id=32184
- Center for Mindfulness. Mindfulness Based Stress Reduction 8-Week Course. http://www.umassmed.edu/cfm/stress-reduction/mbsr-8-week/

## INTERNET ARTICLES/VIDEOS

- Brendel D. There Are Risks to Mindfulness At Work. Harvard Business Review. Available at: https://hbr.org/2015/02/there-are-risks-to-mindfulness-at-work
- Time Magazine: The Mindful Revolution. Available at: http://time.com/1556/the-mindful-revolution/
- Matthieu Ricard: The Habits of Happiness. Available on TED Talks at: http://www.ted.com/talks/matthieu_ricard_on_the_habits_of_happiness?language=en

## MINDFULNESS RESOURCES (cont.)

### BOOKS


- Epstein M. Going to Pieces Without Falling Apart: A Buddhist Perspective on Wholeness. Broadway Books: New York. 1998.
- Harris, Dan. 10% Happier: How I Tamed the Voice In My Head, Reduced Stress Without Losing My Edge, and Found Self-Help That Actually Works—A True Story. Harper Collins: New York. 2014
- Kabat-Zinn J. Full Catastrophe Living: Using the Wisdom of Your Body and Mind to Face Stress, Pain, and Illness. Bantam Books: New York. 2013
- Kabat-Zinn J. Wherever You Go, There You Are: Mindfulness Meditation. Hyperion Books: New York. 1994
- Salzberg S. Real Happiness: The Power of Meditation—A 28-Day Program. Workman Publishing: New York. 2011.
- Sapolsky R. Why Zebras Don't Get Ulcers: The Acclaimed Guide to Stress, Stress-Related Diseases, and Coping, 3rd Ed. St. Martin's Griffin: New York. 2004.
